# Improvement of Shoulder Motion in Two-Stage Dual-Plane Implant-Based Breast Reconstruction followed by Radiation Therapy through Delayed Prepectoral Conversion

**DOI:** 10.1055/s-0043-1775591

**Published:** 2024-02-07

**Authors:** Jin Sol Park, Ung Sik Jin

**Affiliations:** 1 Department of Plastic and Reconstructive Surgery, Seoul National University Hospital, Seoul National University College of Medicine, Seoul, Republic of Korea

**Keywords:** breast reconstruction, acellular dermal matrix, radiotherapy, shoulder

## Abstract

**Background** Although prepectoral implant-based breast reconstruction has recently gained popularity, dual-plane reconstruction is still a better option for patients with poor-quality mastectomy skin flaps. However, shoulder morbidity is aggravated by subpectoral reconstruction, especially in irradiated patients. This study aimed to demonstrate shoulder exercise improvement in subpectoral reconstruction by delayed prepectoral conversion with an acellular dermal matrix (ADM) inlay graft technique at the time of expander-to-implant exchange after irradiation.

**Methods** Patients with breast cancer treated for expander-to-implant exchange after subpectoral expander insertion and subsequent radiotherapy between January 2021 and June 2022 were enrolled. An ADM inlay graft was inserted between the pectoralis major muscle and the previously inserted ADM. The ADM was sutured partially overlapping the pectoralis muscle from the medial side with the transition part, to the muscle border at the lateral side. Perioperative shoulder joint active range-of-motion (ROM) for forward flexion, abduction, and external rotation was also evaluated.

**Results** A total of 35 patients were enrolled in the study. Active shoulder ROM significantly improved from 163 degrees preoperatively to 176 degrees postoperatively in forward flexion, 153 to 175 degrees in abduction, and 69 to 84 degrees in external rotation. There was no difference in patient satisfaction regarding the final outcome between the conventional prepectoral reconstruction group and the study group.

**Conclusion** Shoulder exercises in irradiated patients who underwent subpectoral reconstruction were improved by delayed prepectoral conversion using an ADM inlay graft. It is recommended that subpectoral reconstruction not be ruled out due to concerns regarding muscle contracture and shoulder morbidity in radiation-planned patients with poor mastectomy skin flaps.

## Introduction

Prepectoral implant-based breast reconstruction has recently gained popularity over subpectoral implant-based reconstruction owing to the introduction of an acellular dermal matrix (ADM), which provides an additional soft tissue barrier to the weakened breast skin flap after mastectomy. In prepectoral reconstruction, the pectoralis major muscle is preserved in its anatomical position, resulting in advantages such as reduced pain, shortened hospital stay period, minimization of animation deformity, and reduced capsular contracture. 1 2 Despite the application of ADM, mastectomy flap quality remains one of the most critical factors for successful prepectoral reconstruction. 3 For patients with poor mastectomy skin flap quality or a thin upper pole mastectomy skin flap, subpectoral or dual-plane reconstruction is inevitable. Postmastectomy radiation therapy (PMRT) makes mastectomy skin flaps more vulnerable to wound problems. According to recent meta-analyses, PMRT after prepectoral reconstruction significantly increases wound infection and implant loss rates. 4 5

PMRT is also devastating for subpectoral reconstructions. Radiation-induced tissue toxicity leads to surrounding soft tissue atrophy and fibrosis, intensifying capsular and muscular contractures. It contributes not only to poor aesthetic outcomes but also to irritable functional problems. 6 7 Shoulder morbidity is one of the chief complaints of breast cancer patients, and many studies have observed aggravated shoulder exercise restriction in irradiated patients compared with nonirradiated patients. 8 9 10 11 12 13 Therefore, subpectoral reconstruction is often pushed back by prepectoral reconstruction in the patients planned for PMRT due to anticipated severe capsular and muscular contracture, resulting in shoulder morbidity

If these problems following subpectoral reconstruction and radiation therapy can be solved, do we need to adhere to prepectoral reconstruction? We suggest delayed prepectoral conversion with an ADM inlay graft at the time of expander-to-implant exchange to solve the problems following subpectoral reconstruction in irradiated patients. This study aimed to present a surgical technique that contributes to the improvement of shoulder exercises in irradiated patients who underwent dual-plane reconstruction. To the best of our knowledge, there has been no study which demonstrates the effect of a specific prepectoral conversion surgery technique on the shoulder joint range of motion (ROM).

## Methods

### Study Designs and Patients

A retrospective review of medical records was performed according to the institutional guidelines. The study population included patients with breast cancer who underwent expander-to-implant exchange between January 2021 and June 2022 by a single surgeon (U.S.J.) after immediate postmastectomy tissue expander insertion in the dual-plane. Patients who received PMRT before expander-to-implant exchange surgery and were treated with prepectoral conversion using an ADM inlay graft at the time of expander-to-implant exchange were included. Every patient routinely visited rehabilitation medicine outpatient after mastectomy and got educated on the same home exercise protocol. The patients who received additional physical therapy at rehabilitation medicine clinic were included in the study. Patients were excluded if they underwent radiotherapy before mastectomy, radiation therapy to permanent implants, or additional reconstruction modalities such as latissimus dorsi flap or fat grafting. Patient- and surgery-related characteristics were extracted from electronic medical records. These included age, body mass index (BMI), comorbidities such as diabetes and ipsilateral arm lymphedema, type of mastectomy, axillary treatment, exposure to adjuvant treatments, range and amount of radiation therapy, period between PMRT completion and exchange surgery, pathological staging, inserted ADM type and dimension, inserted implant type, mastectomy tissue mass, and excised skin paddle dimension. The length of follow-up was defined as the period from the placement of a permanent implant to the date of the last shoulder ROM measurement. The institutional review board approved this study design and waived the requirement for informed consent (IRB No. 2210–156–1373).

### Outcome Measures

Active ROM of the affected shoulder joint flexion, abduction, and external rotation was measured by goniometry using a universal manual goniometer a day before the exchange surgery and at postoperative month 1, 6, and 12. Some postoperative measurement days were variable based on the patient's clinic visit days. Passive support was not provided to the arms or shoulders. The starting position of flexion was with the upper and lower arms vertically, and the palm facing the wall behind the patient. The starting position of abduction was with the upper and lower arms vertically, and the palm facing the patient. The starting position of the external rotation was with the upper arm vertically, lower arm horizontally, and palm facing the ceiling. The clinical endpoint of each shoulder exercise was determined when the patient could not move their arm without compensatory movements of the shoulder or trunk. Normal shoulder ROM has been specified by the American Academy of Orthopedic Surgeons as 180 degrees for flexion and abduction and 90 degrees for external rotation. 14

Breast-Q (Reconstruction Module Korean version 2.0) was performed at least 6 months after the placement of a permanent implant in the study group. 15 Breast-Q data were also collected from patients with breast cancer with a history of PMRT who completed immediate two-stage prepectoral reconstruction or conventional two-stage dual-plane reconstruction between January 2020 and June 2022 for comparison with the Breast-Q scores of our study group. The scores of the scales (satisfaction with breasts, satisfaction with implant, psychosocial well-being, physical [chest] well-being) were converted to a value ranging from 0 (worst satisfaction) to 100 (greatest satisfaction).

### Surgical Techniques

**Fig. 1:**
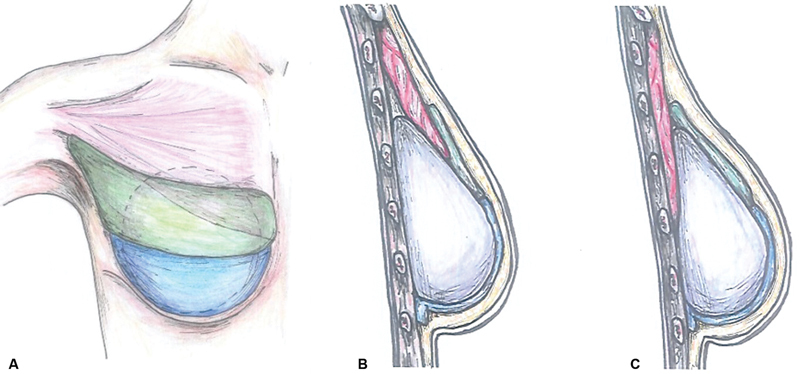
Schematic illustration of the surgical procedure. ( **A** ) An additional acellular dermal matrix (ADM) (green) is laid on the permanent implant as an inlay graft between the previous ADM (blue) and the pectoralis muscle (red) for the prepectoral conversion. ( **B** ) Sagittal view of the breast lateral part. The additional ADM is sutured to the muscle border. ( **C** ) Sagittal view of the breast medial part. The overlapped muscle is placed behind the implant (violet).

**Fig. 2:**
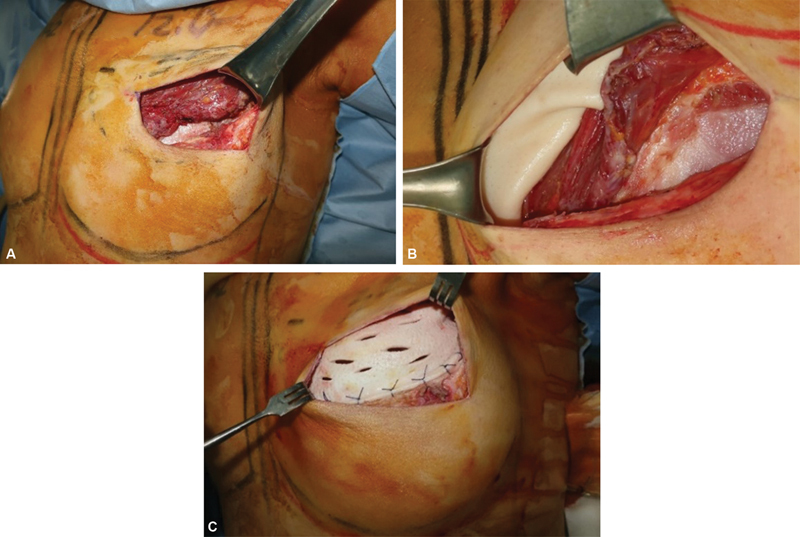
Intraoperative clinical photography. ( **A** ) The incision was made between the pectoralis major muscle and previously inserted acellular dermal matrix (ADM). ( **B** ) The new ADM was sutured to the pectoralis muscle to overlap the medial area of the muscle, and with the transition part, sutured to the muscle border in the lateral area. ( **C** ) The lower border of the additional ADM and the upper border of the previous ADM were sutured to each other.

Delayed prepectoral conversion with an ADM inlay graft was performed at the time of the expander-to-implant exchange. The incision was made between the pectoralis major muscle and previously inserted ADM, and the pectoralis major muscle was detached from the mastectomy skin flap. After tissue expander removal, the breast implant sizer was inserted under the pectoralis muscle and previous ADM. The new ADM, chosen based on the breast base width and mastectomy flap skin thickness, was sutured to the pectoralis muscle to overlap the medial area of the muscle, and with the transition part, sutured to the muscle border in the lateral area. The pectoralis muscle was not fixed to the chest wall. The lower border of the additional ADM and the upper border of the previous ADM were sutured to each other, leaving a gap for implant insertion. Antibiotic solution-irrigated implant insertion was performed using the no-touch technique aided by retractors. After completion of the ADM envelope, the skin incision was closed layer-by-layer in a standard fashion ( Figs. 1 and 2 ).

### Statistical Analysis

Differences between the preoperative and postoperative ROM were analyzed using the Wilcoxon signed-rank test. Univariate and multiple logistic regression analyses were performed for the patient and surgical characteristics to identify factors influencing ROM improvement using 95% confidence intervals. A criterion of *p* < 0.25 was used to determine the inclusion of predictors in the multiple logistic regression model using a backward selection model. Breast-Q scores between groups were analyzed using analysis of variance. The characteristics between the groups were compared using the chi-square or Fisher's exact test for categorical data and the *F* -test or Kruskal–Wallis test for continuous variables. Statistical significance was set at *p* < 0.05. The analyses were performed using SAS 9.4 (SAS Institute, Inc., Cary, NC).

## Results

### Patient Characteristics

A total of 234 expander-to-implant exchange surgeries were performed during the study period, and 35 breasts met the inclusion criteria. The median follow-up duration was 9.0 months (range, 3.2–15.8 months). The mean patient age was 48.9 years (range, 34–72 years) and the mean BMI was 22.2 kg/m ^2^ (range, 18.0–29.3 kg/m ^2^ ). Of the patients, 25.7% received neoadjuvant chemotherapy and 74.3% received adjuvant chemotherapy. Conventional PMRT was delivered at a total of 43.2 Gy in 2.7 Gy fractions over a 4-week period at the department of radiation oncology. The irradiation range included the ipsilateral chest wall, scalene lymph node, internal mammary node, or axillary region, and almost all cases (> 88%) covered at least three of these areas. The median time between the termination of radiation therapy and exchange surgery was 9.6 months. The patient characteristics are summarized in Table 1 , and Table 2 lists the characteristics of the surgical procedures.

**Table 1:** Demographic characteristics of the total 35 patients

Characteristic	Sample ( *N* )	(%)
Age (y)		
Mean 48.9 (8.8) a		
< 50	20	57.1
≥50	15	42.9
BMI (kg/m ^2^ )		
Mean 22.2 (2.5)		
< 18.5	2	5.7
18.5∼22.9	20	57.1
≥23	13	37.1
Pathologic stage		
I or II	22	62.9
III or IV	13	37.1
Diabetes	2	5.7
Ipsilateral upper extremity lymphedema	8	22.9
Radiation dosage		
43.2 Gy/16 fx	27	77.1
> 43.2 Gy/16 fx	4	11.4
Unknown b	4	11.4
Radiation range		
Only chest wall	1	2.9
Chest wall and axilla and IMN	4	11.4
Chest wall and axilla and SCL	3	8.6
Chest wall and IMN and SCL	4	11.4
Chest wall and axilla and IMN and SCL	20	57.1
Unknown b	3	8.6
Period between RTx termination and exchange		
Median 9.6 mo		
< 9.5 mo	17	48.6
≥9.5 mo	16	45.7
Unknown b	2	5.7
Neoadjuvant chemotherapy	9	25.7
Adjuvant chemotherapy	26	74.3
Previous breast surgery		
Ipsilateral BCS	4	11.4

Abbreviations: BCS, breast-conserving surgery; BMI, body mass index; IMN, internal mammary lymph node; RTx, radiotherapy; SCL, supraclavicular lymph node.

a Values are presented as mean (standard deviation [SD]).

b This group includes patients with insufficient medical record from other clinics.

**Table 2:** Operative characteristics of the total 35 patients

Characteristic	Sample ( *N* )	(%)
Type of mastectomy		
NSM	3	8.6
SSM	32	91.4
Type of axillary surgery		
SLNB	16	45.7
ALND	19	54.3
Excised breast mass (g)		
Mean 386.4 (196.1) a		
< 350	19	54.3
≥350	16	45.7
Excised skin paddle dimension (cm ^2^ )		
Mean 73.1 (33.2)		
< 70	15	42.9
≥70	20	57.1
Inserted permanent implant volume (mL)		
Mean 324.7 (84.0)		
< 350	19	54.3
≥350	16	45.7
Immediate ADM dimension (cm ^2^ )		
Mean 86.5 (18.5)		
< 85	19	54.3
≥85	16	45.7
Immediate ADM type		
Alloderm	0	0.0
Bellacell	5	14.3
Cryoderm	21	60.0
Dermacell	5	14.3
Megaderm	4	11.4
Inlay graft ADM dimension (cm ^2^ )		
Mean 62.4 (18.5)		
< 50	17	48.6
≥50	18	51.4
Inlay graft ADM type		
Alloderm	4	11.4
Bellacell	19	54.3
Cryoderm	8	22.9
Dermacell	1	2.9
Megaderm	3	8.6
Postoperative complication b		
Hematoma and capsular contracture	1	2.9
Seroma and capsular contracture	1	2.9
Minor wound problem c	5	14.3
No complication	24	68.6

Abbreviations: ADM, acellular dermal matrix; ALND, axillary lymph node dissection; NSM, nipple-sparing mastectomy; SLNB, sentinel lymph node biopsy; SSM, skin-sparing mastectomy.

a Values are presented as mean (standard deviation [SD]).

b Complications after expander-to-implant exchange surgery.

c It includes skin necrosis and nipple necrosis in the patients who underwent nipple-alveolar reconstruction at the time of expander-to-implant exchange surgery.

Skin-sparing mastectomy was performed in 91.4% of patients, with a mean excised skin paddle dimension of 73.1 cm ^2^ , and axillary resection was performed in 54.3% of patients. Breast prosthesis types were same as Mentor CPX4 breast tissue expander or Mentor MemoryGel smooth round breast implant (Mentor Worldwide LLC, Irvine, CA). The mean dimension of ADM used in the immediate tissue expander insertion and the expander-to-implant exchange was 86.5 and 62.4 cm ^2^ , respectively. The mean volume of the permanent implant was 325 mL, all of which were of the smooth round type. ADM types included AlloDerm (LifeCell Corp., Branchburg, NJ), Bellacell HD (Hans Biomed Crop., Dajeon, Republic of Korea), CGDerm/CGCryoderm (CGBio Corp., Seongnam, Republic of Korea), DermACELL (Stryker Corp., Kalamazoo, MI), and MegaDerm (L&C BIO, Seongnam, Republic of Korea). There were seven patients who received physical therapy at rehabilitation medicine clinic. There were no complications after the expander-to-implant exchange surgery in most patients. Five patients had minor wound problems. Two of them, who underwent nipple-areolar reconstruction at the time of expander-to-implant exchange surgery, experienced nipple necrosis. Three of them experienced partial mastectomy necrosis around the margin of incision site where the pectoralis muscle was detached from the mastectomy skin flap. One patient underwent hematoma evacuation, and another patient underwent revision surgery due to seroma, both of whom resulted in capsular contracture. Some patients complained the newly inserted ADM was noticeable under the thin skin flap.

### Shoulder Exercise Outcomes

**Fig. 3:**
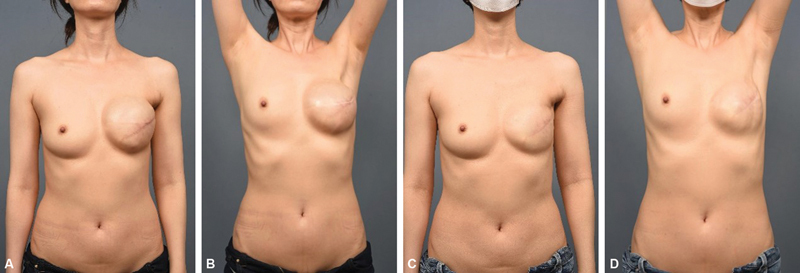
Perioperative photography of a 44-year-old patient. ( **A** , **B** ) A photography 8.5 months after immediate breast reconstruction with tissue expander insertion. ( **C** , **D** ) A photography 9 months after tissue expander to implant exchange surgery. The active forward flexion, abduction, and external rotation improved from 150 to 175 degrees, from 135 to 170 degrees, and from 20 to 90 degrees, respectively.

**Fig. 4:**
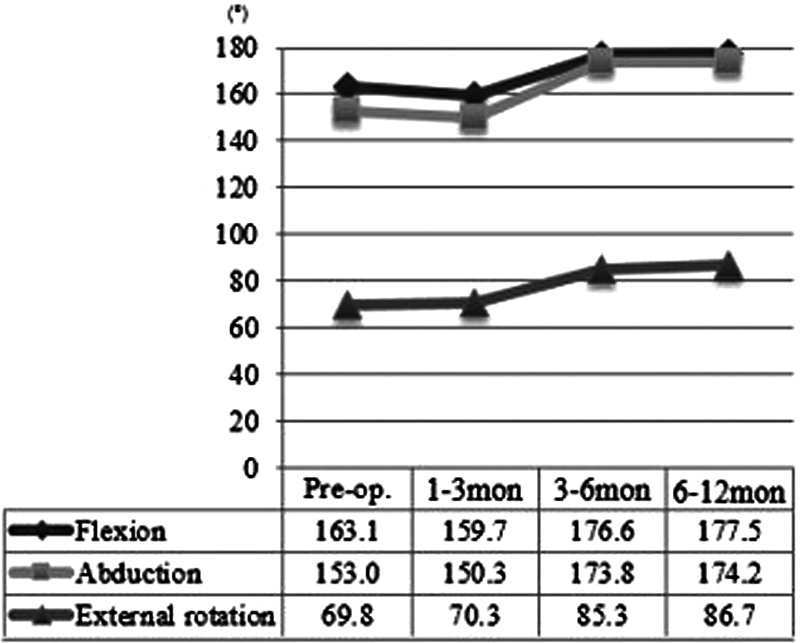
Shoulder range of motion (ROM) sequential change over the period.

Active shoulder ROM significantly improved from 163 degrees preoperatively to 176 degrees postoperatively in forward flexion, from 153 to 175 degrees in abduction, and from 69 to 84 degrees in external rotation based on the last follow-up measurement ( Table 3 and Fig. 3 ). Based on the shoulder ROM progress over the 1-year follow-up period, there was no significant change in ROM 1 to 3 months after surgery, but there was a gradual improvement between 3 and 12 months after surgery ( Fig. 4 ).

**Table 3:** Comparison of shoulder ROM between preoperative and postoperative value

ROM (degrees)	Mean c	Range	Mean	Range	*p* -Value
	Preoperative a		Postoperative b		
Forward flexion	163.06 (23.00)	100–180	176.57 (7.15)	150–180	0.001
Abduction	153.00 (31.81)	90–180	175.71 (9.17)	140–180	< 0.001
External rotation	69.83 (23.33)	10–90	84.43 (11.93)	45–90	< 0.001

Abbreviation: ROM, range-of-motion.

a Preoprative ROM. It was measured 1 day before expander-to-implant exchange.

b Last follow-up ROM after expander-to-implant exchange surgery. It was measured at least 3 months after exchange surgery.

c Mean (standard deviation [SD]).

The extent of ROM improvement was subanalyzed according to patient characteristics (age, BMI, comorbidity of ipsilateral arm lymphedema, and pathological stage) and surgical characteristics (excised breast volume, excised breast skin paddle dimension, axillary dissection status, chemotherapy status, ADM dimension, and permanent implant volume). In the univariate analysis, there were no significant variables, except for age and BMI in shoulder abduction improvement with a *p* -value less than 0.05 ( Table 4 ). After including explanatory variables with a *p* -value less than 0.25 in the univariate analysis, multiple regression analysis showed that sentinel lymph node biopsy (SLNB) rather than axillary lymph node dissection (ALND), lower radiation dose, and longer periods between radiotherapy and exchange surgery significantly increased the extent of flexion improvement. Older age and higher BMI significantly increased the extent of abduction improvement; however, there were no significant variables for external rotation ( Table 5 ).

**Table 4:** Univariate models for shoulder ROM improvement extent

ROM improvement	Forward flexion		Abduction		External rotation	
	Coef. (SE)	*p* -Value	Coef. (SE)	*p* -Value	Coef. (SE)	*p* -Value
Age ≥ 50 (y)	13.10 (7.15)	0.076 c	29.08 (8.48)	0.002 c	12.37 (7. 30)	0.1 c
BMI ≥ 23 (kg/m ^2^ )	6.65 (7.60)	0.388	20.16 (9.48)	0.041 c	13.49 (7.43)	0.079 c
pStage I or II	2.16 (7.68)	0.780	4.93 (10.07)	0.628	2.30 (7.78)	0.769
Ipsilateral arm lymphedema	12.46 (8.57)	0.156 c	–2.71 (11.63)	0.817	13.48 (8.65)	0.129 c
Radiation dose <43.2 (Gy)	–19.82 (9.91)	0.055 c	–13.67 (12.83)	0.295	–10.67 (10.30)	0.309
RTend∼exchange a <9.5 (mo)	17.30 (6.93)	0.018 c	11.05 (9.27)	0.242 c	12.74 (7.46)	0.098 c
ALND	–11.37 (7.19)	0.123 c	–9.39 (9.67)	0.339	–3.04 (7.54)	0.689
Excised skin <70 (cm ^2^ )	5.35 (7.37)	0.473	2.99 (9.76)	0.761	0.82 (7.53)	0.914
Excised breast mass <350 (g)	–8.26 (7.31)	0.267	–0.76 (9.81)	0.939	–10.64 (7.33)	0.156 c
Permanent implant <350 (mL)	–2.49 (7.42)	0.740	3.30 (9.76)	0.737	–0.25 (7.53)	0.974
Grafted ADM ≥62.4 (cm ^2^ )	2.83 (7.41)	0.705	–2.73 (9.76)	0.782	–3.07 (7.51)	0.686
ADM ratio b <0.4	–10.84 (7.19)	0.141 c	–10.43 (9.61)	0.286	1.92 (7.53)	0.800

Abbreviations: ADM, acellular dermal matrix; ALND, axillary lymph node dissection; BMI, body mass index; Coef., coefficient; ROM, range-of-motion; RTx, radiotherapy; SE, standard error.

a Time between radiation therapy completion and expander-to-implant exchange surgery.

b Grafted ADM dimension divided by sum of grafted ADM and immediate ADM dimension.

c *p* -Value < 0.25.

**Table 5:** Multiple regression models for shoulder ROM improvement extent

	Coef. (SE)	*p* -Value
Forward flexion		
ALND	–16.06 (6.07)	0.013
Radiation dose < 43.2 (Gy)	–27.67 (8.30)	0.002
RTend∼exchange a < 9.5 (mo)	17.32 (6.00)	0.007
Abduction		
Age ≥ 50 (y)	30.57 (7.72)	< 0.001
BMI ≥ 23 (kg/m ^2^ )	22.30 (7.91)	0.008

Abbreviations: ALND, axillary lymph node dissection; BMI, body mass index; Coef., coefficient; ROM, range-of-motion; SE, standard error.

Note: Multiple logistic regression analysis of the variables with *p* -value less than 0.25 in univariate regression analysis.

a Time between radiation therapy completion and expander-to-implant exchange surgery.

### Patient-Reported Outcomes

Of the 35 study group patients, 20 completed the Breast-Q questionnaire 6 months after exchange surgery, and their Breast-Q scores were compared with 18 patients who underwent two-stage prepectoral reconstruction and 19 who underwent conventional two-stage dual-plane reconstruction. No differences in characteristics were identified among the three groups, except for age. The converted Breast-Q score (including scales of satisfaction with breasts, satisfaction with implant, psychosocial well-being, and physical [chest] well-being, range: 0–100) of the conventional dual-plane group was 50.0 ± 23.0, that of the study group was 53.3 ± 19.9, and that of the prepectoral group was 54.6 ± 19.0, but there were no significant differences between the groups. Higher scores correspond to higher satisfaction. The mean score of postradiotherapy well-being scale of Breast-Q questionnaire, in which lower scores correspond to higher satisfaction (range: 6–18) was 9.9 ± 2.5 in the study group, 10.2 ± 2.0 in the prepectoral reconstruction group, and 10.6 ± 2.3 in the conventional dual-plane reconstruction group, but there were no significant differences between the groups ( Table 6 ).

**Table 6:** Patient-reported outcome

	Study group	Prepectoral group a	Dual-plane group b
Converted Breast-Q score c	53.3 (19.9)	54.6 (19.0)	50.0 (23.0)
Postradiation well-being scale score d	9.9 (2.5)	10.2 (2.0)	10.6 (2.3)

Note: Values are presented as mean (standard deviation [SD]).

a Conventional two-stage prepectoral reconstruction.

b Conventional two-stage dual-plane reconstruction.

c It includes scales of satisfaction with breasts, satisfaction with implant, psychosocial well-being, and physical (chest) well-being. Higher scores correspond to higher satisfaction; range: 0–100.

d Lower scores correspond to higher satisfaction; range: 6–18.

## Discussion

This study demonstrates that delayed prepectoral conversion with the ADM inlay graft technique at the time of expander-to-implant exchange improves shoulder joint active ROM compared with the status before exchange surgery in breast cancer patients who underwent immediate breast reconstruction with tissue expander insertion in the dual-plane and radiation therapy after mastectomy. There was no significant difference in patient satisfaction regarding the final outcome of the reconstruction between the conventional prepectoral reconstruction group and the study group.

The introduction of ADM has brought about the advent of dual-plane reconstruction and further progress in mastectomy and reconstruction techniques such as refined ablative procedures, perfusion imaging technology, stabilized implants, fat grafting, and several implant pocket formation techniques compensated for complications related to prepectoral reconstruction, opening a new era of prepectoral reconstruction. 16 In prepectoral implant placement, the lack of dissection of the pectoralis muscles results in the absence of animation and a more satisfactory breast contour with more natural ptosis. Furthermore, studies comparing prepectoral and subpectoral reconstruction have shown similar rates of overall perioperative complications such as hematoma, seroma, wound necrosis, and wound infection, even in the setting of PMRT. 1 17 18

However, most of these studies state selection bias as a limitation of their study and mastectomy skin flap quality is still regarded as an essential element for the success of prepectoral reconstruction where less vascularized soft-tissue coverage is performed over the implant. Comorbidities, such as uncontrolled diabetes or morbid obesity, history of tobacco use, high-grade breast ptosis, and postoperative radiation therapy are known risk factors of prepectoral reconstruction. They are thought to compromise microvascular circulation of skin flap and surrounding soft tissue, leading to increased risk of skin flap necrosis and implant exposure. 3 19 In a recent meta-analysis, PMRT was also demonstrated to significantly increase the rate of wound infection in prepectoral reconstruction. 4 5 However, patients who are expected to receive postoperative radiation are usually regarded as more appropriate for prepectoral reconstruction, considering radiation-induced pectoralis fibrosis and distortion in subpectoral or dual-plane reconstructions.

PMRT has been more widely applied as a radiation modality, with evidence of benefit in survival. However, it reversely raises the risk of complications associated with implants, resulting in reconstruction failure and burnout. Radiation produces free radicals, reactive oxygen species, transforming growth factors, and inflammatory cytokines, which impair the recovery and repopulation of stromal stem cells. The basal layer is chronically repopulated by the proliferation of surviving clonogenic cells. Furthermore, injured vascular endothelial cells are insufficient to maintain normal microvascular blood supply. In the acute phase of irradiation toxicity, the skin exhibits erythema and dry or moist desquamation. In the later phase, telangiectasia, dermal fibrosis, discoloration, and atrophy of the glands appear in the skin. These responses result in wound dehiscence, infection, necrosis, and delayed healing in PMRT settings. The skin and surrounding soft tissue changes present as pectoralis fibrosis, followed by animation deformity, severe degree of capsular contracture, and distortion of the breast contour. 7 20 21 22

Recently, prepectoral conversion has emerged as an effective solution to address the disadvantages of both prepectoral and subpectoral reconstructions, as mentioned above. Prosthesis pocket position changes from the submuscular plane to the prepectoral plane have been demonstrated to improve chronic breast pain and animation deformity. 23 24 Surgical techniques of 239 prepectoral conversion studies included in a systematic review by Maria et al described the fixation of the inferior border of the dissected pectoralis muscle to the posterior capsule or to the chest wall with or without capsulectomy. 24 ADMs were employed for anterior implant coverage or complete implant coverage, except for three studies. However, no prepectoral conversion study has performed anchorage of the inferior border of the dissected pectoralis muscle to the newly inserted ADM, rather than to the chest wall. In addition, no prepectoral conversion study has presented shoulder ROM progress or focused on patients receiving PMRT. To the best of our knowledge, this is the first study to analyze the effect of delayed prepectoral conversion surgery using the ADM inlay graft technique and depict objective shoulder function improvement, focusing on irradiated patients.

Shoulder morbidity is one of the chief complaints of patients with breast cancer, and chronic arm or shoulder discomfort is known to last up to 3 years after breast surgery. Postmastectomy patients experience 5.7 times higher postoperative shoulder problems than postbreast-conserving surgery patients. Anatomical modifications and substantial reduction in shoulder function after mastectomy have been analyzed in several studies, and shoulder flexion and abduction ROM showed up to an 18 and 45% decrease, respectively, at 1 month postoperatively. Patients who underwent ALND or PMRT showed greater aggravation in shoulder ROM and strength compared with the SLNB or nonirradiated groups. A considerable percentage of breast cancer survivors who return to their workplace are unable to work full-time because of physical limitations, including shoulder problems. It seems essential to improve patients' shoulder movement to increase the return-to-work rate and quality of life. 25 26 27 Patients who undergo both subpectoral reconstruction and PMRT are more prone to severe shoulder morbidities, which decrease their quality of life. 8 More delicate management is needed to reduce shoulder discomfort, particularly in irradiated patients who undergo subpectoral reconstruction after mastectomy.

According to other studies on shoulder morbidity after mastectomy, shoulder ROM decreases until the first postoperative month and shows gradual improvement until postoperative month 12, when it reaches a plateau. 25 26 27 28 Even though our study focused on the shoulder ROM change after tissue expander-to-implant exchange surgery, not mastectomy, the postoperative shoulder ROM progress in our study is similar to that of other studies, as shown in Fig. 4 . To our knowledge, no study has analyzed shoulder ROM progression after tissue expander-to-implant exchange surgery.

In our study, the common variables that influenced the extent of shoulder ROM with a significance level of 25% in the univariate analysis were age and the period between radiotherapy and exchange surgery. This may be in line with many other studies that have demonstrated a positive correlation between the degree of capsular contracture and age or time after irradiation. 29 30 Older patients seem to have more severe capsular contracture than younger patients, and radiotherapy-induced capsular contracture tends to aggravate over time owing to radiation-induced irreversibility. Therefore, appropriate surgical intervention is critical for patients with more progressive fibrotic changes. In addition, the ratio of the inlay grafted ADM dimension to the entire ADM dimension influenced the extent of shoulder flexion, but not abduction or external rotation, with a significance level of 25%. This may be related to the fact that the pectoralis major, which is mainly manipulated in this surgery, plays a major role in shoulder flexion rather than abduction or external rotation. 31

Many studies have demonstrated higher Breast-Q scores in the prepectoral reconstruction group than in the dual-plane reconstruction group, which is consistent with the results of our study. Although the difference was not statistically significant, there was a tendency for patients to be more likely to satisfy conventional prepectoral reconstruction or prepectoral conversion reconstruction rather than conventional dual-plane reconstruction. Our study showed that patients' quality of life and subjective satisfaction with reconstruction may be improved by delayed prepectoral conversion with an ADM inlay graft as high as conventional prepectoral reconstruction group patients. Noticeably, postradiotherapy well-being scale tended to be higher in our study group than the conventional prepectoral or dual-plane reconstruction group, although this was not statistically significant. It is speculated that this lack of significance is due to the small study population.

This study had some limitations. This study was based on the experience of a single surgeon at a single institution with a small study population and lacks long-term follow-up data. A large-scale study with a long-term follow-up is promising. In addition, the preoperative ROM value depends on rehabilitative exercise and patients' efforts, so we focused on the extent of improvement. Also, there is a possibility of influence of overinflation of tissue expander compared with permanent implant volume on ROM measurement improvement. However, the mean discrepancy between the volume of final expander and the volume of implant was 70 mL, and there was no significant relation between the extent of ROM improvement and the extent of volume discrepancy. Finally, there is no data on shoulder movements in patients with prepectoral reconstruction. However, the purpose of this study is to confirm whether shoulder problem in subpectoral reconstruction can be improved by this approach. We intended to identify whether the final general outcome of this approach is comparable to that of prepectoral reconstruction, so, we compared Breast-Q scores between them.

In conclusion, shoulder exercise in irradiated patients who underwent subpectoral reconstruction was improved by delayed prepectoral conversion using an ADM inlay graft, and patient satisfaction with the reconstruction tended to be as high as that of patients with conventional prepectoral reconstruction. Our results are in line with previous studies that demonstrated that delayed prepectoral conversion is safe and improves patient satisfaction. Even though PMRT after dual-plane reconstruction has a critical effect on shoulder morbidity and dual-plane reconstruction is not the priority compared with prepectoral reconstruction in the setting of PMRT, considering the availability of prepectoral conversion at the time of exchange surgery, employment of dual-plane reconstruction should not be avoided in radiation-planned patients. Dual-plane reconstruction can be a better option for patients with devastating quality mastectomy skin flaps who are planned for PMRT if appropriate surgical intervention is possible, as in our study.
